# SCAPULAR FRACTURES: COMPARISON OF GLENOPOLAR ANGLE MEASUREMENT USING 3D COMPUTED TOMOGRAPHY AND CONVENTIONAL RADIOGRAPHY

**DOI:** 10.1590/1413-785220263403e300508

**Published:** 2026-06-12

**Authors:** João Artur Bonadiman, Patrick Thiago Minski, Felipe Damasceno Appel, Eduardo Bervian, Paulo Piluski, Osvandré Lech

**Affiliations:** 1 Hospital Sao Vicente de Paulo, Instituto de Ortopedia e Traumatologia, Passo Fundo, RS, Brazil.; 2 Instituto Brasil de Tecnologias da Saude, Rio de Janeiro, RJ, Brazil.; 3 Clinica Kozma, Passo Fundo, RS, Brazil.

**Keywords:** Bone Fracture, Scapula, Shoulder Fractures, Observational Study, Reproducibility of Results, Fratura, Escápula, Fraturas do Ombro, Estudo Observacional, Reprodutibilidade dos Testes

## Abstract

**Objective:**

To compare the interobserver and intraobserver reliability of glenopolar angle (GPA) measurement in scapular fractures using conventional radiography (2D) and three-dimensional computed tomography (3D-CT).

**Methods:**

This retrospective observational study included 33 patients with fractures of the scapular body or neck treated between 2016 and 2023. Anteroposterior radiographs and 3D-CT scans obtained at the time of diagnosis were included. The GPA was measured by eight independent evaluators, and data were analyzed using the intraclass correlation coefficient (ICC), according to the criteria proposed by Koo and Li, with SPSS software.

**Results:**

The mean interobserver ICC for GPA measurement was 0.959 (95% CI: 0.934–0.977) for radiography and 0.971 (95% CI: 0.953–0.984) for 3D-CT, indicating excellent reliability for both methods. Individual intraobserver reliability ranged from 0.598 to 0.869, with five evaluators demonstrating good reliability and three showing moderate reliability. The sample consisted predominantly of males (93.9%), with a mean age of 39.6 years (range: 19–78 years), and 24.2% of patients underwent surgical treatment.

**Conclusions:**

GPA measurements obtained by radiography and 3D-CT demonstrated high interobserver reliability, with slight superiority of 3D-CT. Both methods are valid, although 3D-CT may be preferable in cases requiring greater angular precision. ***Level of Evidence: III; Retrospective observational study.***

## RESUMO

**Objetivo:**

Comparar a confiabilidade inter e intraobservador da aferição do ângulo glenopolar (AGP) em fraturas da escápula utilizando radiografias convencionais (2D) e tomografias computadorizadas com reconstrução tridimensional (TC-3D).

**Métodos:**

Estudo observacional retrospectivo conduzido com 33 pacientes com fraturas do corpo ou colo da escápula atendidos entre 2016 e 2023. Foram incluídas radiografias anteroposteriores e TC-3D obtidas no momento do diagnóstico. O AGP foi aferido por oito avaliadores independentes e os dados foram analisados por meio do coeficiente de correlação intraclasse (CCI), conforme os critérios de Koo e Li, utilizando o software SPSS.

**Resultados:**

A média do CCI interobservador para a aferição do AGP foi de 0,959 (IC95%: 0,934–0,977) para a radiografia e de 0,971 (IC95%: 0,953–0,984) para a TC-3D, indicando excelente confiabilidade para ambos os métodos. A confiabilidade intraobservador individual variou de 0,598 a 0,869, com cinco avaliadores apresentando boa confiabilidade e três com confiabilidade moderada. A amostra foi composta majoritariamente por homens (93,9%), com média de idade de 39,6 anos (19–78 anos), sendo 24,2% tratados cirurgicamente.

**Conclusões:**

As aferições do AGP por radiografia e TC-3D demonstraram alta confiabilidade interobservador, com leve superioridade da TC-3D. Ambos os métodos são válidos, sendo a TC-3D preferível em casos com necessidade de maior precisão angular. ***Nível de evidência: III; Estudo comparativo retrospectivo.***

## INTRODUCTION

The scapula is a flat bone that connects the upper limb to the axial skeleton, providing stable support for muscular action and serving as a dynamic base in maintaining glenohumeral kinematics.^1^ Fractures of this bone are rare, accounting for about 1% of all fractures, with approximately 22% of these fractures resulting from high-energy trauma and 21% presenting at least one associated fracture, reinforcing their association with severe clinical conditions.^2^ The treatment of these fractures aims to preserve shoulder function and prevent complications such as misalignments that lead to degenerative changes, subacromial impingement, and scapular dyskinesia. Among the criteria used to evaluate fractures of the body and neck of the scapula, the measurement of the glenopolar angle (GPA) stands out, often considered in the decision between conservative and surgical treatment.^3,4^

**Figure 1:**
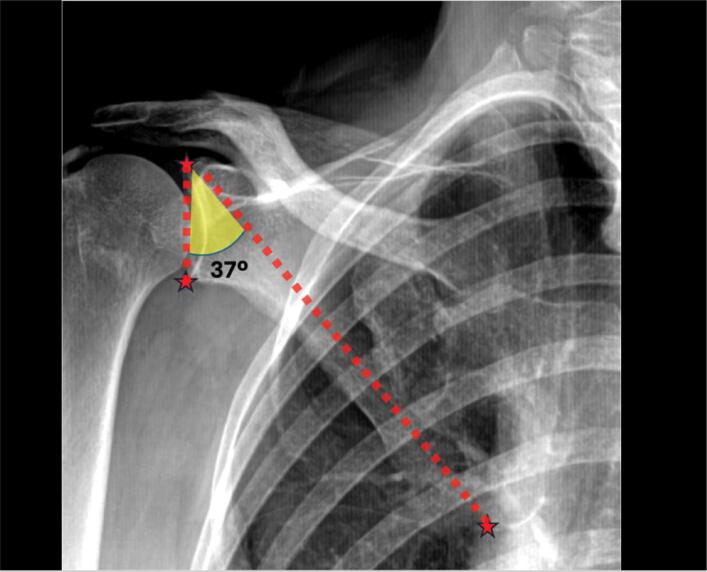
Measurement of the glenopolar angle in a true AP shoulder radiograph.

The concept of the GPA is defined by the angle between two lines: one drawn from the inferior pole to the superior pole of the glenoid, and another from the superior pole of the glenoid to the inferior angle of the scapula (Figure 1), with normal values ranging between 30° and 45°.^5,6^

Romero et al.^7^ applied the GPA in the evaluation of fractures of the neck of the scapula, demonstrating that significant deviations in this angle were associated with worse clinical outcomes. Since then, the GPA has become an important parameter in the decision-making process regarding surgical treatment of scapular fractures, especially those involving the neck and body of the scapula.

Traditionally, this measurement is performed using radiographs. However, in environments such as emergency rooms for polytraumatized patients, radiographs are often obtained in inadequate positions or with non-standard projections, which can compromise the accuracy of the measurement and directly impact therapeutic conduct.

In light of this condition, recent studies suggest that computed tomography with three-dimensional reconstruction (CT-3D) yields less inter- and intra-observer variability in measuring the GPA than conventional radiography.^8–10^ Therefore, this study aims to compare the inter- and intra-observer reliability of GPA measurement in fractures of the neck and body of the scapula, using radiographs and CT-3D. Our hypothesis is that the measurements performed by CT-3D exhibit greater reproducibility and accuracy than those obtained with conventional radiography.

## MATERIALS AND METHODS

An observational retrospective study was conducted, in which medical records and imaging exams of patients with fractures of the body or neck of the scapula (ICD-10: S42.1), treated at a single institution between the years 2016 and 2023, were analyzed. The project was approved by the institution's Research Ethics Committee under number CAAE 73769523.8.0000.5342.

Patients with fractures of the body or neck of the scapula were included, regardless of the treatment performed (surgical or conservative), provided they had an anteroposterior radiograph of the scapula and a CT-3D obtained at the time of diagnosis. Patients who did not undergo both imaging exams, those with isolated fractures of other portions of the scapula (such as the acromion, spine, or coracoid process), cases diagnosed late (more than three weeks after the trauma), images of low quality that hindered adequate angular measurement, and individuals who did not consent to participate in the study were excluded.

**Figure 2:**
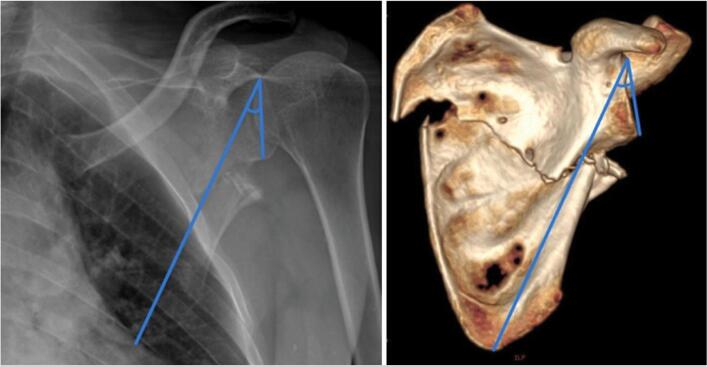
Measurement of the glenopolar angle in an AP radiograph taken in the emergency room and in a CT-3D scan of the same patient.

After screening, the images were randomized and analyzed by eight independent evaluators (orthopedic surgeons). The evaluators were previously instructed on the measurement method and the parameters used. For standardization, the anteroposterior incidence of the shoulder was used as a radiological reference, and in CT-3D, the reconstruction of the posterior face of the scapula in the plane of the greatest mediolateral diameter was used. (Figure 2)

The GPA measurements were recorded in a spreadsheet (Microsoft Excel, Redmond, WA, USA) and subsequently submitted to statistical analysis using SPSS (Statistical Package for the Social Sciences) software. The intraclass correlation coefficient (ICC) was used to assess measurement reliability across different observers and imaging methods, following the guidelines proposed by Koo and Li.^11^ (Table 1)

**Table 1:** Intraclass correlation coefficient (ICC) according to Koo & Li et al.

Range of CCI	Reliability Classification
< 0.50	Low reliability
0.50 – 0.75	Moderate reliability
0.75 – 0.90	Good reliability
> 0.90	Excellent reliability

## RESULTS

Radiographs and CT-3D scans of 33 scapula fractures were evaluated (Table 2). Regarding the epidemiology of the sample, 31 patients (93.9%) were male and 2 (6.1%) were female, with an average age of 39.6 years (ranging from 19 to 78 years) at the time of the trauma. In terms of laterality, 18 fractures (54.5%) affected left scapulae and 15 (45.5%) affected right scapulae. Regarding treatment, 8 patients (24.2%) underwent surgical treatment, while 25 (75.8%) received conservative treatment.

**Table 2:** Assessment of the GPA of 33 patients (P) by 8 evaluators (AV).

Measurement of the Glenopolar Angle																
	AV1		AV2		AV3		AV4		AV5		AV6		AV7		AV8	
	RX	TC3D	RX	TC3D	RX	TC3D	RX	TC3D	RX	TC3D	RX	TC3D	RX	TC3D	RX	TC3D
P1	24	27.1	22	25.9	20.3	29.9	24.9	26.5	29	39.7	17.1	30.5	24.9	32	25.1	29.2
P2	34.5	37.4	21.5	38.6	22.3	37	29.5	41.8	24.2	30.1	17.8	39.1	13.6	40.1	48.4	39.3
P3	22.4	25.6	23.9	25.3	23.9	30.3	21.2	32.6	22.4	31.2	36.5	35.5	26.9	34.4	31.6	31.4
P4	24.1	15.1	18.1	7.3	16.9	13.4	19.7	16.9	21.1	13.8	23.2	18	17.5	17.3	17.8	13.8
P5	38.5	38.4	39.6	34	43.5	39.4	35	37.5	39.3	37.1	44.6	40.4	36.4	39.5	37.9	40
P6	14.7	24.1	22	18	17.2	32.2	17.9	22.7	17.9	33.8	20	24	18.8	24	15.6	34.1
P7	43.8	43	44	41.4	40.5	39.2	43.4	47.5	40.8	48.4	35.3	45.3	35.5	48	41.2	46.3
P8	32.8	30.5	32.1	30.2	31.3	33.3	25.8	31.6	30.1	32.4	29.8	31.8	26.2	32.3	30.3	32
P9	41	39.9	40.3	38.5	45.3	42	42.1	42.2	41	44.2	43.6	41.1	37	46	48.5	47.7
P10	21.9	30.7	22	28.5	20.6	32.1	22.3	30	21.5	29.6	16.3	30.6	15.9	32.4	21.8	30.2
P11	43.7	47.8	41.5	48.5	47.9	33.5	48.5	41.8	45.8	47	52	45.6	49.8	36.7	62	52.5
P12	22.1	34.5	30.2	30.6	25.2	27.9	18.6	33.9	22.5	33.6	33.1	20.3	36.3	31.1	20.7	32.4
P13	23.1	26.5	13	17.3	25.5	19.2	16.9	24	18.7	16.4	21.2	16.9	20	30.4	22.7	16.1
P14	24	18.4	17.1	12.1	30.1	18.9	22.7	19.6	23.7	19.4	23.3	20.2	23.7	22.8	33.3	17.8
P15	36.8	39.8	30.9	28.8	16.5	39.9	31.3	41	30.4	45.6	36.1	45.2	37.7	40.3	38	44.6
P16	23.1	26.4	27.34	45.3	23.4	29.8	21.6	42.4	40.7	29.7	20.1	28.7	29.5	32	32	28.2
P17	32.9	33.6	34	28.5	34.3	35.8	33.8	36.4	32.8	37	37.9	37.3	37.4	37.3	31.5	41.3
P18	32.5	31.2	25.7	41.5	43.1	32.9	30.6	38.5	17.4	35.6	34.7	42.3	43.1	24.9	31.5	37
P19	38.3	47.4	45.9	40.5	42.5	51.5	43.9	50.3	39.9	46.8	45.9	47.8	44	48	49.2	49.3
P20	18.3	32.4	22.6	25.6	27.6	29.2	24	27.8	26.8	29.9	18.2	30.9	21.7	32.9	43.7	29.6
P21	23	27.8	9.5	19.3	15.3	28.7	27	29.1	16.6	29.9	23.8	29.1	15.1	29.4	17.4	28.6
P22	45	45.5	47.8	44.3	42.6	44.6	40.3	43.5	42	46	41.2	44.4	42.6	45.8	45.2	40.7
P23	26.5	40.8	24.9	28.9	26.3	32.2	25.2	41.2	23	44.2	32.6	50	26.5	42.8	29.7	35.2
P24	29.4	26.8	35.4	32.5	31.2	27.4	26.3	25.8	23.4	25.3	28.5	26.4	35.8	30.3	38.6	26.7
P25	36.1	29.1	34.2	30	32.6	33.9	31.3	33.1	39	35.8	31.1	33.8	34.4	35.1	37.2	30.6
P26	44.8	33.6	36.3	31.5	42.4	33.4	40.4	34.5	40.6	37.8	31.6	40.5	33.6	36	43.1	36.5
P27	27.4	35.3	19.4	23.8	20.8	33.5	25.8	34	24.2	34.9	19.9	38.7	25.9	33	25.5	38.2
P28	26.8	27.6	24	25.6	26.6	29.2	21.3	25.4	24.3	25.8	27.3	24.3	30.6	23.3	31.8	24.3
P29	45.7	42.5	48.5	45.2	46.1	38.1	39.2	45.9	44.6	43	44.9	48.7	37.3	43.4	46.1	42.5
P30	21.3	28.1	21.2	25	25.4	27.5	26.4	25.5	24.5	26.9	20.8	27.7	32	32.2	24.4	29.9
P31	35	37.4	36.3	32.5	31.2	37.7	33.2	35.7	32.5	38.8	34.3	38.5	33.8	39.6	34	37.7
P32	30	32.1	33.5	32.9	31.5	29.7	30.6	30	30.2	30.5	32	33.9	33.9	27.3	36.8	35.8
P33	35.2	43.6	39.4	48.4	40.4	44.4	34.2	47.9	35.7	46.1	41.7	50.8	38.3	46.4	43.6	52.8

Regarding individual variability, we obtained intraclass correlation coefficients (ICCs) ranging from 0.598 to 0.869, as shown in Table 3.

**Table 3:** Individual intraclass correlation coefficient of the evaluators.

Evaluator	Mean Value Intraclass Correlation	95% Confidence Interval	
		Lower Limit	Upper Limit
AV1	0.826	0.635	0.915
AV2	0.869	0.736	0.935
AV3	0.68	0.365	0.841
AV4	0.772	0.305	0.907
AV5	0.733	0.337	0.881
AV6	0.766	0.465	0.891
AV7	0.598	0.201	0.799
AV8	0.805	0.604	0.904

Table 3. Individual intraclass correlation coefficient of the evaluators.

After analyzing the intraclass correlation coefficient of all evaluators, we obtained an average value for GPA measurement by radiograph of 0.959 (0.934 – 0.977) and GPA measurement by CT-3D of 0.971 (0.953 - 0.984), where "1.0" (absolute/hypothetical) would be the perfect measurement without variability between methods or among evaluators (Table 4).

**Tabela 4:** Coeficiente de correlação intraclasse comparativo entre RX e TC-3D.

Método de imagem	Valor médio correlação intraclasse	Intervalo de confiança 95%	
		Limite inferior	Limite superior
Radiografia	0,959	0,934	0,977
TC-3D	0,971	0,953	0,984

We observed that both measurement methods have excellent reliability. According to Koo & Li^11^, five of our evaluators achieved good individual reliability in their measurements. The other three evaluators achieved only moderate individual reliability in their measurements. When analyzing the class correlation coefficient by summing the individual measurements, both imaging methods showed good reliability, indicating low interobserver variability.

## DISCUSSION

The therapeutic plan for scapular body fractures can be guided by the criteria described by Cole et al., who recommend surgical treatment in the presence of lateral translation >2 cm, angulation >45° in the lateral view, fractures associated with the clavicle or the acromioclavicular joint, and GPA <22°.^9,12,13^ Therefore, the precise measurement of GPA plays a critical role in defining the conduct.

In our study, excellent interobserver reliability was observed for GPA measurements using both evaluated methods, with a slight superiority of CT-3D over conventional radiography (CCI: 0.971 vs. 0.959). These findings corroborate the results presented by Anavian et al.^14^, who found CT-3D more reproducible for angular measurements of the scapula, and by Suter et al.^10^, who demonstrated less interference from rotation in the evaluation of GPA with three-dimensional reconstructions.

It is important to consider that, in emergency situations involving polytraumatized patients, obtaining radiographs may be performed under suboptimal conditions, with variations in positioning that compromise the accuracy of GPA measurement. Tadros et al.^15^ and Wijdicks et al.^16^ demonstrated that rotational deviations can significantly alter the value of GPA, directly impacting the therapeutic indication. Hong et al.^17^ reinforced this finding by reporting an average difference of 6.1° between values obtained on radiographs and those on CT-3D.

Labronici et al.^18^ demonstrated that scapular rotation of up to 30° – whether internal or external – can substantially reduce the value of GPA, reinforcing the recommendation to measure the scapula in a neutral position. In the present study, the variation of CCI among evaluators (0.598 to 0.869) suggests the influence of technical familiarity and individual experience, highlighting the importance of standardization in measurement.

Regarding functional outcomes, Bi et al.^19^, in a review of 669 patients with extra-articular scapular fractures, reported good functional results with both surgical and conservative approaches. Similarly, Morey et al.^20^, in a review of floating shoulder treatment, did not identify significant functional differences between therapeutic modalities but observed that restoration of GPA was associated with better clinical scores.

This study presents as its main strengths the methodological standardization in image analysis, the use of multiple independent evaluators, and the direct comparison between two widely used methods in clinical practice for GPA measurement. Additionally, the inclusion of a representative sample of patients treated in a trauma setting enhances the applicability of the results. On the other hand, this is a retrospective study, with inherent limitations of this design, such as dependence on the quality of archived images and possible selection bias. The absence of correlation with clinical or functional outcomes also represents a limitation, preventing the extrapolation of findings for direct prognostic impact. Prospective studies, with functional follow-up and control of clinical variables, are necessary to validate and expand the conclusions presented here.

Finally, although the CT-3D has shown slightly superior reliability, it is necessary to consider its availability, cost, and radiation exposure. When obtained under appropriate conditions, X-rays remain a valid tool for measuring the GPA. Based on our findings, we recommend the complementary use of CT-3D in cases of diagnostic uncertainty or when the therapeutic decision depends on more precise angular measurements.

## CONCLUSION

The measurements of the GPA by X-ray and CT-3D demonstrated high interobserver reliability in evaluating fractures of the body and neck of the scapula. Both methods showed low variability among evaluators, with a slight superiority of the CT-3D, which presented a slightly higher intraclass correlation coefficient.

These findings reinforce the use of CT-3D as a tool for greater precision, especially in cases with borderline surgical indications. Standardization of measurement techniques and training of professionals are essential to ensure consistency in evaluations. New studies that associate radiological parameters with clinical and functional outcomes may contribute to more informed and individualized therapeutic decisions.

## Data Availability

Research data will be available upon request from reviewers.
